# Response of Cucumbers (*Cucumis sativus* L.) to Waste Wood Fiber Substrates and Additional Nitrogen Fertilization

**DOI:** 10.3390/plants11243464

**Published:** 2022-12-10

**Authors:** Rita Čepulienė, Lina Marija Butkevičienė, Lina Skinulienė, Vaida Steponavičienė

**Affiliations:** Department of Agroecosystems and Soil Sciences, Vytautas Magnus University, K. Donelaičio Street 58, 44248 Kaunas, Lithuania

**Keywords:** cucumber productivity, chlorophyll index, substrate mixtures, growing media

## Abstract

As the consumption of plant products grown under regulated-climate conditions intensifies, its production also intensifies. Peat substrate as a growing medium is widely used due to its desirable physical, chemical, and biological properties. Peatlands play an important ecological, economic, and cultural role in human well-being, but their resources are decreasing, so wood fiber can be used as a substitute for peat substrate. Cucumbers (*Cucumis sativus* L.) were cultivated in growing media (Factor A) of peat substrate and wood fiber: (1) peat substrate (PS); (2) wood fiber (WF); (3) WF and PS 50:50 *v*/*v*; or (4) WF and PS 25:75 *v*/*v*. To evaluate the amount of additional nitrogen, four fertilization rates were used (Factor B): (1) conventional fertilization (CF); (2) N_13_; (3) N_23_; or (4) N_30_. The yield of cucumbers grown in wood fiber depended on the amount of additional nitrogen. When plants were fertilized with the highest rate of additional nitrogen, N_30_, their yield increased by 22% compared to the yield of cucumbers that were grown in wood fiber using conventional fertilization. Chlorophyll synthesis was the most intense in the leaves of cucumbers grown in wood fiber when the plants were additionally fertilized with N_23_ and N_30,_ and in mixtures of wood fiber with peat substrate in ratios of 50:50 and 25:75, fertilized with N_23_ and N_13_, respectively. In summary, it can be argued that cucumbers can be grown in wood fiber and in a mixture with peat substrate in a ratio of 50:50, but additional nitrogen is needed to compensate for the amount of nitrogen immobilized in the fiber.

## 1. Introduction

In recent years, the development of greenhouse vegetable production has become one of the most important vegetable production facilities in the world due to its fast and efficient use of solar radiation and its ease of management and production yield [1]. Greenhouse plants are usually grown in vegetative containers filled with substrates. Peat is the main substrate component used in greenhouses in the US and Europe [2,3] and typically accounts for 50–80% of the total substrate volume [4,5]. The remaining 50–20% is usually made up of secondary materials such as perlite, lignin, mineral wool [6,7], vermiculite, sand, coconut fiber, or pine bark. Peat substrates are widely used due to their desirable physical, chemical, and biological properties [8]. This material has a low bulk density, a high porosity, and chemical properties that can be easily tailored by the grower to meet the needs of the plant [9]. However, peat substrate is made from peat from drained peatlands, and this resource is limited. Wetlands have been drained to be converted into agricultural land, fuel, or industrial and urbanized areas. More than half of the world’s wetlands have already been lost [10]. This is destroying the ecosystem from a sink to a net source of greenhouse gases in the land use sector [11,12]. The novelty of our investigation is the modeling and assessment of the nitrogen amount according to the physiological state of the plants; the influence of additional compensatory nitrogen on cucumber biometric, photosynthetic, and productivity parameters; and variations in the nitrogen, carbon-to-nitrogen ratio, and electrical conductivity of the growing media.

Soilless cultures generally refer to any method of growing plants that does not use mineral soil in the growing media [13], which includes hydroponics and growing media/substrates of any origin. Compared to outdoor production, vegetables grown without mineral soil (soilless) can have yields that are 2–5 times higher than conventional outdoor yields, while using 10 times less water and having year-round yields, better flavor, and a higher nutritional value. This has led to a significant increase in the cultivation of soilless crops in recent years worldwide [14]. The medium in which plants grow is a living organism with a continuous cycle of nutrient metabolism, where microorganisms break down organic residues of plant and animal origin into simpler organic compounds and mineral substances (ions) through complex biochemical processes, which become readily available plant nutrients [15].

The choice of growing media (substrate) is one of the most important factors for the growth of regulated-climate crops. Substrates used in greenhouses vary in origin, with some being artificially produced (mineral wool) and others derived from natural products (peat substrate, coconut fiber, aged pine bark, perlite, volcanic rock, tuff, expanded clay pellets, vermiculite, zeolite, pumice, and sand) [16,17,18,19].

There is a growing interest in restoring degraded peatlands and finding alternative materials to replace peat substrates used in agriculture. Alternatives to peat include tree bark and wood fiber, which have many of the properties of peat, with the exception of a lower water retention capacity and therefore a higher air capacity. The specific properties of composts strongly influence their potential as a fertilizer [20]. Wood fiber, a waste product from the wood-processing industry, can replace peat and coir in growing media [21]. It has phytosanitary properties [22] and can act as a carrier for biocontrol organisms due to the activity of microorganisms [23], but has a lower nitrogen uptake by plants [24]. Researchers in the Netherlands chose five different organic substrates (*Sphagnum* peat, media pine bark, coarse pine bark, coconut fiber, and wood fiber) for cucumber cultivation in order to compare them with perlite. Summarizing the results, they concluded that all organic substrates could provide the same or an even higher yield and quality of cucumber fruit compared to perlite in a Dutch hydroponic system [19]. Other researchers, in order to investigate the problem of a lack of microorganisms in the substrate and to solve the problem of spreading pathogens with a preventive system, infected substrates with *Trichoderma* spp. Four substrates were evaluated: a mixture of 100% peat; 30% wood fiber and 70% peat; 30% wood fiber and 70% peat; and a mixture of 50/50%. The substrates were tested with three varieties of *Juniperus communis*. Different combinations of wood fiber and peat in the growing media gave better results compared to only the peat media. Summarizing the results, the researchers stated that wood fiber is a suitable alternative to peat when adding 30% and/or 50% to the growing media [25]. 

Increasing the consumption of regulated-climate crops leads to production that is more intensive. For the intensive greenhouse production of vegetables, berries, herbs, and flowers, the most common growing media is mineral wool. However, it is not an environmentally friendly substrate and must be disposed of. Finding a suitable location for new landfills is becoming increasingly more difficult and is no longer a popular solution. Researchers’ results have shown that cucumbers grown on date palm waste had significantly higher yields compared to those grown in soil, but there was no significant difference compared to those grown in perlite [26]. Wood fiber could be an alternative to mineral media for growing plants in industrial greenhouses. The advantage of wood fiber as a growing media is that it is biodegradable and does not need to be recycled (as with mineral wool). It can be composted, used as mulch, and used to improve the soil in gardens and flowerbeds. Wood pulp, a recycled wood waste, has great potential as an alternative to peat in soilless substrate mixtures [27]. 

The chemical properties of the growing media, such as the pH, electrical conductivity, and many others, are equally important when growing plants in vegetative containers under regulated-climate conditions. All these chemical properties interact and change continuously due to the small volume of the growing media, which is limited by the vegetative container. Therefore, this study aims to gain new insights into the variation in the carbon/nitrogen ratio of wood fiber during the microbial degradation of the fiber, the nitrogen demand during the cucumber-growing period, and how this influences the growth and development of the plants. The uneven process of fiber destruction, which changes the nitrogen demand, complicates the technology of growing plants in wood fiber growing media under regulated-climate conditions. The problem is the appropriate and timely selection of nitrogen during the cucumber-growing season to compensate for the nitrogen used for wood fiber decomposition. The aim of this study was to generate new knowledge for the development of a technology to compensate for nitrogen immobilization during wood fiber destruction by growing plants on wood fiber alone and in combination with a peat substrate. The novelty of the investigation was the modelling and evaluation of the nitrogen content according to the physiological state of the plants; the influence of additional compensatory nitrogen on cucumber biometric, photosynthetic, and productivity parameters; the variation in the carbon–nitrogen ratio; and the variation in the electrical conductivity of the growing medium.

## 2. Results 

### 2.1. Biometric Parameters, Yield of Cucumber Plants, and Productivity

When the cucumber biometric parameters were evaluated in different growing media and at different nitrogen fertilization rates, the best development was observed when the plants were grown on PS, compared to plants grown on other growing media without additional compensatory nitrogen fertilization, using conventional fertilization (CF) (Table 1). Plants grown on WF were 1.6 times shorter at the beginning of flowering than plants grown on PS and 1.4 times shorter than plants grown on both WF and PS mixtures. The plants were significantly shorter, by 24%, at the beginning of flowering when they were grown in PS with N_23_ nitrogen fertilization compared to the height of the plants grown without additional fertilization. The height of the cucumbers grown in WF and in WF/PS mixtures 27 days after transplanting was not significantly affected by the additional nitrogen fertilization, although the highest rate of N_30_ tended to stimulate vegetative growth.

The assessment of the number of flowers on the plants 27 days after transplanting showed that the number of flowers on PS was 1.25 times higher than on the WF/PS 25/75 mix, 2.5 times higher than on the WF/PS 50/50 mixture, and 3.0 times higher than on the WF mix. The number of flowers on cucumbers on the WF mixture was on average 5.0 to 13.0 times lower than on the other tested media, but the flowering of the WF cucumbers was enhanced by an additional nitrogen fertilization. Increasing the nitrogen rate increased the number of flowers on the plant, whereas without additional fertilization, the WF cucumbers did not flower yet. The largest difference between the additional N fertilization was found on WF/PS 25/75 plants, with 25% fewer flowers on plants fertilized with N_30_ compared to the number of flowers on plants that were not additionally fertilized.

Throughout the growing season, the plants produced stable fruit on the WF/PS 50/50 medium, and the highest number of fruits was found when the plants were fertilized with 1.24 to 1.29 times additional N_13_ and N_23_ compared to the other media. Excessive nitrogen (N_30_) in the peat substrate and in WF/PS 25/75 reduced the cucumber productivity. The average number of fruits per cucumber plant grown in PS decreased with an increasing nitrogen rate, to N_23_ and N_30_, compared to N_13_ and without additional fertilization. Cucumbers grown in WF with N_13_ and N_23_ fertilizers had an increased number of cucumber fruits per plant, by 8.0% and 22.0%, respectively, compared to cucumbers grown without N fertilizer. The fruit weight of cucumbers grown on the WF medium decreased from 4.5 to 16.3% with increasing rates of additional N compared to WF without additional N. On the WF/PS 50/50 medium, with different rates of additional N, the fruit weight was found to be the highest in comparison to the other media studied, ranging from 1.09 to 1.32 times higher, and the additional N fertilization increased the weight of the cucumbers on the plant, especially with the rate N_13_. On the WF/PS 25/75, an additional N fertilization reduced the weight of the cucumbers on the plant by between 7.0 and 21.1% compared to conventional fertilization. 

The evaluation of the different rates of additional nitrogen in different growing media showed that 27 days after transplanting, the highest dry matter accumulation was observed for the WF medium compared to the other growing media (Table 1). The change was more pronounced with the addition of N_30_, as the dry matter content was found to be between 1.02 and 1.12 times lower than in the other media at the same nitrogen rate. At the beginning of cucumber fruiting, the dry matter accumulation of plants grown in the wood fiber medium was the lowest, and at the highest nitrogen rate, N_30_, the dry matter content of the aboveground part of the cucumber was reduced on average by a factor of 1.2 to 1.45 compared with the other growing media studied. From the beginning of plant maturity, the plants grown on wood fiber media accumulated the least dry matter at higher nitrogen rates. This trend continued throughout the growing season. In all phases of the study, the application of a conventional fertilizer to the wood fiber media resulted in a higher level of dry matter in the aboveground part of the plants than in the plants that were additionally fertilized with compensatory nitrogen. 

### 2.2. Chlorophyll Index of Cucumber Leaves

**Figure 1:**
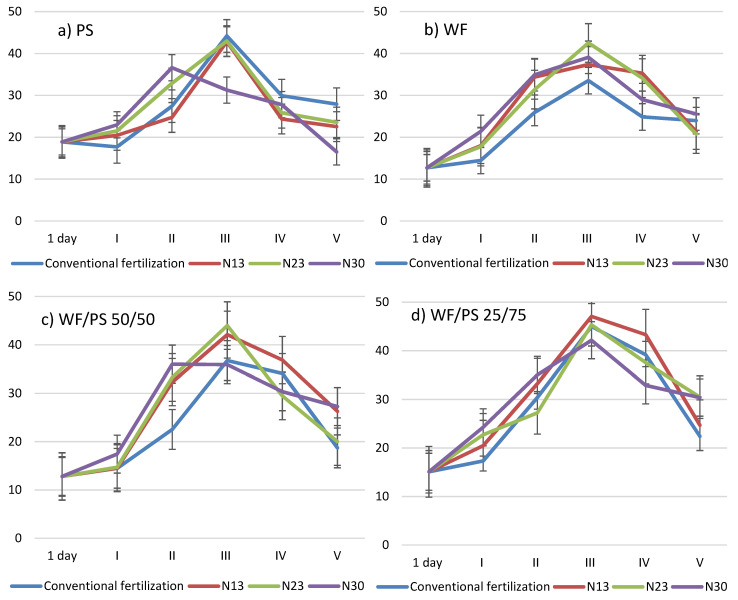
The influence of growing media and additional nitrogen fertilization on the chlorophyll index in cucumber leaves during the growing season; 1 day—1 day after transplanting; I–V—five experimental stages: 27, 39, 52, 64, and 80 days after transplanting of cucumbers into the growing media. (**a**) PS—peat substrate; (**b**) WF—wood fiber; (**c**) WF/PS 50/50—mixture of wood fiber and peat substrate 50:50 *v*/*v*; and (**d**) WF/PS 25/75—mixture of wood fiber and peat substrate 25:75 *v*/*v*. N13, N23, and N30—additional nitrogen fertilization; Vertical bars indicate the mean error SE.

The chlorophyll index (CHI) in cucumber leaves was the highest during the intensive cucumber fruiting in all the media studied (Figure 1). Cucumbers grown in peat substrate with N_30_ fertilizer showed an increase in the CHI 27 days after transplanting, but a decrease at later stages compared to cucumbers grown with the lower rates of N_13_ and N_23_ and without supplemental N. Comparing the nitrogen rates used in the WF growing media, the most intense CHI was found in the leaves of cucumbers grown with the highest rates of additional nitrogen, i.e., N_23_ and N_30_. In the WF/PS growing media, the CHI was less intense when N_30_ was applied.

### 2.3. Electrical Conductivity in Growing Media with Additional Nitrogen Fertilization

**Figure 2:**
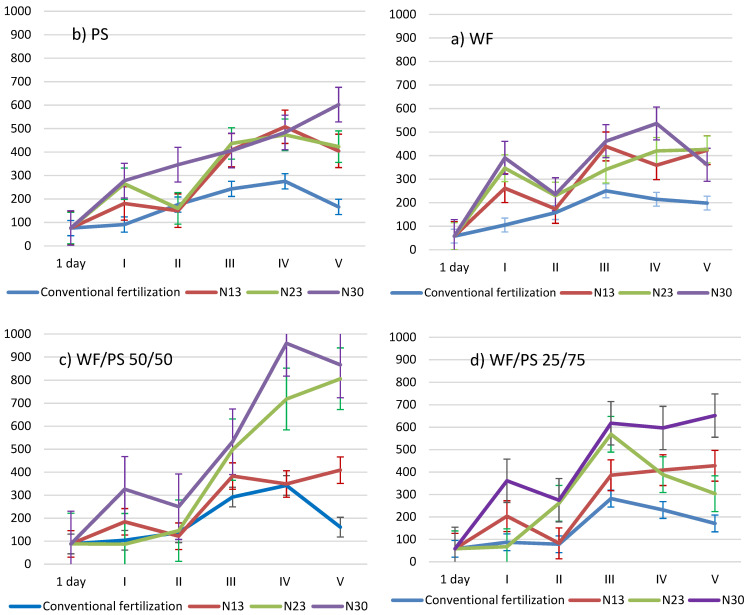
The influence of additional nitrogen fertilization on the variation in electrical conductivity (m S m^−1^) in growing media during the cucumber-growing season; 1 day—1 day after transplanting; I–V—five experimental stages: 27, 39, 52, 64, and 80 days after transplanting of cucumbers into the growing media. (**a**) PS—peat substrate; (**b**) WF—wood fiber; (**c**) WF/PS 50/50—mixture of wood fiber and peat substrate 50:50 *v*/*v*; and (**d**) WF/PS 25/75—mixture of wood fiber and peat substrate 25:75 *v*/*v*. N13, N23, and N30—additional nitrogen fertilization; Vertical bars indicate the mean error SE.

The electrical conductivity describes the concentration of all salt ions dissolved in the solution of the growing medium. The electrical conductivity of the growing media is determined by a number of factors—the material of the media itself, the concentration of fertilizer (salts), the irrigation water, and the level of leaching, depending on the irrigation method chosen [4]. The curves of electrical conductivity in the growing media show a downward slope from the beginning of cucumber flowering (stages I to II) and an upward slope during cucumber fruiting (stages III and IV) (Figure 2). It can be assumed that nitrogen immobilization is most intense at this time. The most intense immobilization of nitrogen took place in the wood fiber. In this growing media, the electrical conductivity decreased by 88 m S m^−1^ with the additional nitrogen fertilizer N_13_, by 111 m S m^−1^ with N_23_, and by 155 m S m^−1^ with the highest nitrogen rate of N_30_. In the WF/PS 50/50 growing medium, the electrical conductivity decreased by 62 m S m^−1^ from the beginning of flowering (stage I) to the beginning of fruiting (stage II) with the additional N_13_ application and by 76 m S m^−1^ with the nitrogen fertilization rate of N_30_. The electrical conductivity decreased by 121 and 86 m S m^−1^ in the WF/PS 25/75 growing media. 

Under conventional fertilization of cucumbers, the electrical conductivity in the wood fiber media increased to up to 250 m S m^−1^ during stage III and decreased at the end of the growing season (stage V). Similar trends in electrical conductivity during the cucumber-growing season were observed in the other growing media: the peat substrate increased to 275 m S m^−1^ at the peak of cucumber fruiting; the 50/50 media increased to 349 m S m^−1^ at the peak of cucumber fruiting; and the 25/75 medium increased to 282 m S m^−1^ at the peak of cucumber fruiting (stage III). In all four cucumber-growing media tested, the electrical conductivity was fertilizer-dependent and was the highest when cucumbers were fertilized with additional N_30_.

### 2.4. Total and Mineral Nitrogen Content of Growing Media under Additional Nitrogen Fertilization

**Figure 3:**
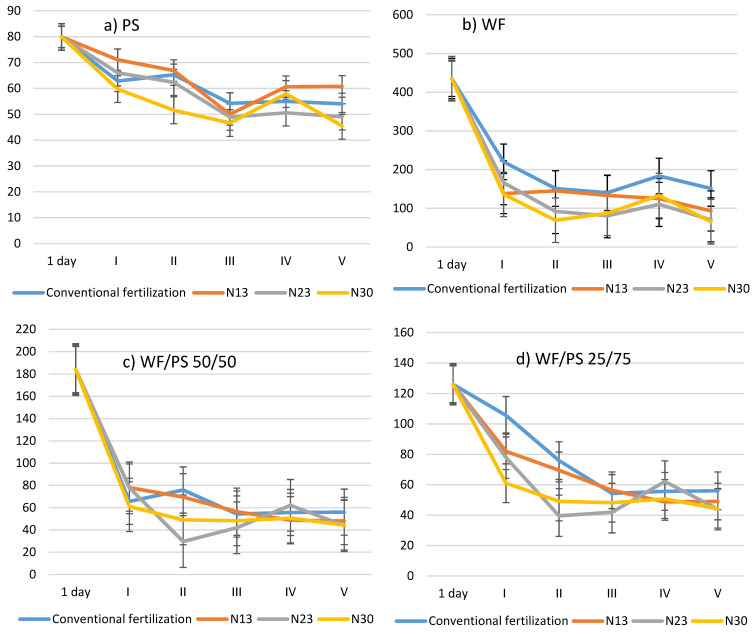
The influence of additional nitrogen fertilization on the variation in the carbon-to-nitrogen ratio (C:N) in growing media during the cucumber-growing season; 1 day—1 day after transplanting; I–V—five experimental stages: 27, 39, 52, 64, and 80 days after transplanting of cucumbers into the growing media. (**a**) PS—peat substrate; (**b**) WF—wood fiber; (**c**) WF/PS 50/50—mixture of wood fiber and peat substrate 50:50 *v*/*v*; and (**d**) WF/PS 25/75—mixture of wood fiber and peat substrate 25:75 *v*/*v*. N13, N23, and N30—additional nitrogen fertilization; Vertical bars indicate the mean error SE.

At the beginning of the experiment (stage I), the peat substrate had the highest total nitrogen (N_total_) content, while the WF/PS 50/50 and 25/75 mixtures had a lower, but non-significantly lower, N_total_ content (Figure 3). The WF had the lowest levels of this macronutrient compared to the other media. In the first stage of the study, the addition of N_30_ to the cucumbers significantly increased its content in WF and both mixtures of WF/PS compared to the media without additional fertilization. In the WF substrate, the addition of nitrogen significantly increased the N_total_, with a 2.6-fold increase from N_13_ and N_30_ and a 4.2-fold increase from N_23_. The addition of N_23_ to the PS substrate increased the N_total_ by 8.3%, and N_30_ increased it by 16.7%. In the WF/PS 50/50 and 25/75 growing media, the increase in N_total_ was only due to the addition of N_30_, which increased it by 13.7 and 36.8%, respectively. 

In stages II and III, the N_total_ content increased in all growing media, even without an additional nitrogen application. This could indicate that nitrogen immobilization into the matrix of the growth media was the most intense during this period. N_13_ fertilization did not affect the N_total_ content compared to the media without additional fertilization (conventional fertilization), with the exception of the WF media. In the latter, N_13_ fertilization resulted in a 39.2% decrease in the N_total_ content in stage II, while increasing the N rate to N_23_ resulted in a significant increase in the N_total_ content not only in the wood fiber media, but also in both WF/PS mixtures. The most significant increase in the N_total_ in the growing media was due to the additional N_30_ fertilization and, compared to the N_total_ content in the media without additional fertilization, the N_total_ content was higher (in the PS—23.1%, in the WF—41.8%, in the 50/50—28.7%, and in the 25/75—42.4%). 

Under intensive cucumber fruit growth (stage IV), a decrease in the N_total_ content in the media was observed. The N_total_ content of the PS growing media was not significantly different at any fertilization rate compared to the unfertilized substrate. In the WF media, the N_total_ content was significantly lower compared to the PS and WF/PS 50/50 and 25/75 mixtures, and additional fertilization increased the N_total_ content of the media. At the end of the cucumber-growing season (stage V), the N_total_ content was significantly (53.3%) higher in the PS media where the highest compensatory nitrogen rate, N_30_, was applied. Lower supplementary rates resulted in a marginal increase in the N_total_ content compared to substrates without compensatory nitrogen. In the WF media, all the additional nitrogen rates tested significantly increased the N_total_ content by between 2.0 and 2.7 times. In the WF/PS mixtures, the lowest rate of N_13_ had no effect on the N_total_ content of the growing media at the end of the growing season, but when the rate was increased to N_23_ and N_30_, the N_total_ content of the growing media was significantly higher than in the media without supplementary fertilization and with a rate of nitrogen of N_13_.

The additional nitrogen fertilization applied to the plants during the study increased the mineral nitrogen (N_min_) content in all growing media (Table 2). At 27 days after transplanting (stage I), the additional fertilization with N_13_ resulted in a significant increase in the mineral nitrogen only in the WF/PS 50/50 media by a factor of 22, while in the other media, the increase was insignificant in comparison with the mineral nitrogen content in the media without additional fertilization. In all the growing media studied, the N_min_ content of mineral nitrogen increased significantly with the addition of N_23_ and N_30_ compared to the N_min_ content of the media with addition fertilization and to the N_min_ content of the control media, PS, with conventional fertilization. This trend of N_min_ content in the growing media was also observed 80 days after transplantation (stage V).

The fertilization of cucumbers grown in wood fiber (WF) media with all the levels of additional nitrogen tested significantly increased the mineral nitrogen content of the media, compared to the WF media with conventional fertilization, where N_min_ was significantly lower than in the control PS growing media with conventional fertilization. In WF/PS 50/50 and WF/PS 25/75, the application of additional nitrogen N_23_ and N_30_ resulted in significantly more mineral nitrogen in these growing media, both 27 and 80 days after transplanting cucumbers, compared to the N_min_ in the media where conventional fertilization was applied.

### 2.5. Carbon-to-Nitrogen Ratio (C:N) in the Growing Media of Cucumbers with Additional Nitrogen Fertilization

The carbon-to-nitrogen ratio (C:N) tended to decrease in all growing media during the cucumber-growing season (Figure 3). The lowest variation was observed in the peat substrate without additional fertilization or with a nitrogen rate of N_13_. The drop in C:N in the WF growing media and in mixtures with peat (WF/PS 50/50 and WF/PS 25/75) during the first 27 days after transplanting (stage I) was significant.

In the later stages of the study, the C:N ratio varied insignificantly in all the media studied, with a more intense variation with higher rates of additional nitrogen—N_23_ and N_30_. For WF/PS 50/50, the decrease in C:N was also observed in the first phase of the study and did not change significantly thereafter. The change in the WF/PS 25/75 C:N was not as sharp as in the WF medium and the WF/PS 50/50 mixture, but the trend was similar. In the WF media and in the WF/PS mixtures, the decrease in C:N was more pronounced with the addition of N_23_ and N_30_, while with the application of the lowest rate of compensatory nitrogen, N_13_, the decrease in C:N was similar to that observed in the media without additional compensatory nitrogen fertilization.

## 3. Discussion

The use and post-use disposal of synthetic substrates is a global issue, and peat substrates are currently particularly popular as a result of the destruction of an important part of the ecosystem, peatlands [11,12]. Therefore, ways to substitute for peat and synthetic media are being sought. In our experiment, cucumbers were selected as nutrient-demanding plants to be grown in vegetative containers in order to replace the peat substrate (or part of the substrate) with wood fiber. There are published results showing that cucumber fruits, grown in a greenhouse with wood fiber, accumulated more nutrients than those grown in other organic media [19]. In studies where perlite was mixed with leaf compost and sludge, it was found that the use of different growing media alone or in combination significantly increased the development and productivity of cucumbers, with particularly good results obtained with the combination of leaf compost + perlite + sludge (1:1:1). The researchers explained that the results were due to the high organic matter content of the substrate mixtures, which significantly improved the nutrient uptake by cucumbers [28]. The leaf area of the plants showed better vegetative growth of the cucumbers in wood fiber, but in a pine bark substrate, the cucumber yield was higher and the fruit quality was better compared to those grown in perlite. The finer fraction of the pine bark media resulted in a higher cucumber yield and a higher vitamin C content of the fruit by 23.9% and 48.3%, respectively, compared to cucumbers grown in perlite. In the large fraction of pine bark growing media, the cucumber yields were similar to perlite, but the quality was higher [19]. In our experiment, 27 days after transplanting the seedlings in WF media, the fresh weight of the plants was lower, they were shorter, and they produced fewer flowers and leaves, but they had a higher dry matter weight compared to those grown in PS and WF/PS mixtures (Table 1). The results of the study showed that excessive nitrogen rates, N_23_ and N_30_, had a negative effect on cucumber productivity. How plants accumulate dry matter is important for their development. A high accumulation of dry matter indicates that the plant is not getting enough or getting too many nutrients. In a stressful situation, the plant tries to protect itself by storing nutrients. The biometric parameters of cucumbers grown in the WF/PS 25/75 mixture were similar to those of cucumbers grown in PS. Scientists who have carried out studies with synthetic substrates have confirmed that all organic substrates are better than synthetic substrates in terms of vegetative and reproductive growth. Cucumbers grown in perlite showed poorer growth in both the vegetative and reproductive phases: the leaves showed signs of nutritional deficiency, the fresh and dry matter of the vegetative part was reduced, and the yields were lower. Scientists have explained that this phenomenon is due to an indirect effect of substrates on nutrient retention and availability [5,29]. In the last century, similar observations have been made showing that the quality and quantity of tomato fruits grown in organic media were better than those grown in inorganic media [30]. Organic substrates were found to contain higher levels of nitrogen and phosphorus, but the plant less readily takes up potassium in organic substrates than perlite. Wood fiber (WF) media also has a nutrient retention characteristic due to the continuous destruction processes that take place [15,31]. Cucumbers grown in peat had higher fresh and dry weights of the vegetative fraction, but the earliest and more abundant fruits were produced in the finer fraction of pine bark. The fresh fruit yields were higher in the peat–wood fiber mixture [5]. In our experiment, cucumbers grown in the WF/PS 25/75 mixture without additional fertilizer produced significantly more fresh fruit (Table 1). In the PS media and in the WF/PS 50/50 mixture, the highest fruit yields were obtained only with additional N_13_ fertilizer, while in the WF media, the highest rate of additional N_30_ increased the cucumber yield. The highest cucumber fresh weight was obtained in WF/PS 25/75, but only with additional N. Our experimental results, similar to those of other researchers, show that synthetic and peat substrates can be substituted for wood fiber, but that the nutrient balance is very important [29]. 

Plant growth and yield performance are often dependent on the N supply, and it has been shown that the optimal N content is within a certain range, with cucumber yields increasing with increasing N content. However, yields start to decrease when the N content exceeds the limit [32]. At the beginning of the growing season, 27 days after transplanting the seedlings, the additional nitrogen fertilization with WF had no significant effect on the plant height, number of flowers, or number of leaves, but the lowest additional rate of N_13_ significantly increased the plant’s aboveground mass. Additional N_23_ and N_30_ in PS resulted in a significant decrease in the plant height, whereas in the WF/PS 25/75 substrate, only additional N_30_ decreased the plant height and in the WF/PS 50/50, it increased it. An additional N application to the PS and WF/PS 25/75 mixture resulted in fewer leaves, whereas only higher N rates in WF and WF/PS 50/50 increased the number of leaves per plant (Table 1). In contrast, the lowest N_13_ rate in all growing media increased the aboveground weight of the plant, while increasing the N rate in the media was similar to that in the media without additional fertilization. The dry matter content of cucumbers at the end of the growing season was significantly increased by additional N_23_ and N_30_ in the mixed growing media and the WF substrate, whereas in the PS media, only the additional N_23_ increased the dry matter weight. The results obtained only confirmed the researchers’ claims that cucumber development starts to decline when nitrogen levels are exceeded [32]. The irrigation rate, N, and their interaction significantly influenced the weight per fruit, with higher N and irrigation rates being beneficial for increasing the yield up to a certain limit. The partial factorial productivity of the available N uptake increased gradually with decreasing N content [33]. It has been shown that plant growth and yield performance are often dependent on N supply. An adequate NO_3_-N supply from adequate N fertilization increases the amount and activity of nitrate reductase; this in turn increases the NO_3_-N reduction potential and provides a greater capacity for total amino acid synthesis, protein synthesis, or total N assimilation [34]. 

The chlorophyll content and distribution in leaves are important indicators of plant nutritional information. In the WF growing media, only the highest N_23_ and N_30_ increased the chlorophyll index, whereas in PS and both mixtures, these values were the lowest (Figure 1). This could indicate that nitrogen immobilization into the matrix of the growth media was the most intense during this period. The chlorophyll index values increased up to 52 days after transplanting (stage III), whereas in PS, they increased up to stage II, and only after the addition of N_30_. Chlorophyll is one such compound in leaves that is strongly influenced by the nitrogen content of the plants [35,36]. The chlorophyll index value in plant leaves is also an indicator of an N deficiency, as concluded by researchers in a study with different cucumber chipping rates and densities in a vegetative container [37]. The optimal plant development and the highest yield can be achieved by monitoring chlorophyll values. Chlorophyll values provide information about adjustments and applications of N fertilizer to improve the performance of fertilized cucumber seedlings [38]. The replacement of peat with pine wood fiber did not affect the leaf chlorophyll index, the growth of shoots, the plant height and width, the substrate N, or the shoot tissue N percentage at the end of production. However, petunias grown on peat fiber substrates retained dark green foliage with high leaf chlorophyll index values (≥44.4) and more flowers per plant [31]. Better results were obtained for the leaves, leaf mineral substances, chlorophyll concentrations, and yield in plants grown in leaf compost + perlite + sludge (1:1:1) media [28]. Studies have shown that chlorophyll measurements are directly related to the N content of vegetable diets, and significant differences have been obtained in crops [39,40]. 

The use of wood fiber is a good alternative to synthetic substrates, but there is a problem with N immobilization. Wood recycling waste products used as substrates can immobilize fertilizer nitrogen and influence the development of potted plants [41]. In a similar study with petunias, the aim was to assess the effect of wood fiber substrates on nutrient levels in plant tissues and plant development, with a focus on assessing N immobilization [31]. Substrates consisting of peat and hammer-milled pine (*Pinus* sp.) wood (peat:wood) or coconut (*Cocos nucifera*) fiber (peat:coconut) were used for the comparison, while 100% peat substrate was used as the control. Previous studies have shown that when plants are grown in a media with wood fiber without additional fertilizer, their growth and quality begin to deteriorate rapidly. It has been shown that with more than 30% wood fiber in the growing media, the cultivation technology needs to be adjusted [31]. In our experiment, by monitoring the variation in N in the growing media with wood fiber during the cucumber-growing season by increasing the rate of N, it was found that additional rates of compensatory nitrogen, N_13_ and N_23_, did not increase the N_total_ content in the media (Table 2). Only increasing the rate of additional N_30_ fertilization significantly increased the N_total_ content in the media. Conventional fertilization of cucumbers in the wood fiber at the beginning of the growing season increased the amount of N_total_, with little variation at later stages. In the mixtures, all additional rates of compensatory N intensively increased the N_total_ content of the substrate up to stage IV, whereas in stage V, the N_total_ content was only increased by increasing the fertilization to N_13_ and N_23_. Researchers investigating nutrient leaching in greenhouse substrates indicated that nutrient removal rates varied between substrates depending on the growth stage of the plants: all nutrient removals were significantly lowest in perlite and wood fiber, except for N_total,_ where wood fiber removal was highest, probably due to N immobilization in the wood fiber [42]. Peat, in contrast, consistently removed the most nutrients, except for the N_total_, which was lower than the bark and wood fiber values of 20.5% and 22.7%, respectively, but higher than perlite at 19.4%. At the flowering stage, only the amount of the N_total_ leaching showed a significant difference between the treatments. Perlite had the lowest N_total_ leaching, while bark-M had the highest value [19].

Nitrogen is only available to plants in the form of mineral compounds, with the main sources being ammonium and nitric acid salts. Plants more readily take up ammoniac nitrogen when the calcium, potassium, and magnesium are sufficient. Phosphorus and molybdenum facilitate the uptake of nitrate nitrogen. The additional compensatory nitrogen applied to the plants during the study increased the mineral nitrogen content during the growing season in all growing media. When fertilized with N_13_ at the lowest level of compensatory nitrogen, the increase in mineral nitrogen was significant in the peat substrate by a factor of 7.6; in the mixture of wood fiber with peat substrate PS/WF 50/50, it increased by a factor of 22; and in the mixture PS/WF2 5/75, the increase was insignificant compared with the mineral nitrogen content in these media at the beginning of the experiment (Table 2). The results of studies conducted by other researchers on the proportion of wood fiber in the substrate, ranging from 10% to 40%, in the cultivation of several species of Pelargonium showed that ornamental plants can be successfully cultivated on a peat substrate containing 20% waste wood fiber and supplemented with nitrogen, while the substrate containing 40% wood fiber had a negative effect on all the parameters of growth and the leaf content of macro- and micronutrient uptake [29]. 

Different substrates contain substances that can have direct and/or indirect effects on plant growth and development. The selection of a well-balanced substrate with a variety of materials is essential to obtain good, stable plant yields [26]. The supply of nitrogen (N) is crucial for the formation of amino acids, proteins, nucleic acids, and other cellular components. Fertilization, which includes organic residues, depends on an efficient rate of N mineralization, as the mineral forms (nitrate and ammonium) are more readily taken up by plants [43,44]. The nitrogen balance is not only important in artificial media, but also natural media, with carbon-to-nitrogen ratios above 30 tending to mobilize nitrogen due to the intense microbial decomposition of carbon, which consumes nitrogen. In wood fiber, the carbon-to-nitrogen ratio exceeds 300 [4]. In our experiment, the C:N ratio in the wood fiber (WF) media was 450. Additional fertilization with higher rates of N_23_ and N_30_ in particular reduced the C:N ratio to 80, although without additional fertilization, it decreased to 150 in the WF media. In peat, the C:N ratio was the lowest at 80 and tended to decrease with increasing fertilizer rate until the end of the cucumber vegetation. In WF and in the PS/WF mixture, the C:N ratio decreased most intensively during the first stage of the experiment (up to 27 days after transplanting) (Figure 3). When C sources are abundant in the media, large amounts of N can be immobilized in the microbial biomass [23]. Other researchers [19] have shown that wood fiber removed the highest amount of N from the cycle throughout the growing season, but removed lower amounts of other types of nutrients than other organic substrates. Such growing media immobilizes N and reduces the amount of N available to the plants and their ability to take up N. Already in the last century, it was recognized by researchers that the rate-limiting step in N assimilation, the reduction of NO_3_-N to nitrite NO_2_-N, which is catalyzed by nitrate reductase, is highly regulated [45]. Nitrogen immobilization is one of the key factors regulating both the synthesis and activity of nitrate reductase. Deposits of N-containing organic compounds are important sources of mineralizable N [41] and many recent studies have shown the importance of direct uptake by roots into the total N and C budget of plants [46,47,48,49].

## 4. Materials and Methods

### 4.1. Site Description and Materials of Growing Media 

The vegetation experiment was carried out at Vytautas Magnus University Agriculture Academy, Joint Research Centre of Agriculture and Forestry in a regulated-climate greenhouse on 7 June 2021. Two substances were chosen for the cucumber-growing medium: wood fiber and peat substrate. The wood fiber was produced by the Lithuanian company Nereta (a subsidiary of JSC Rekyva) from the highest-quality wood chips (residues from the wood industry). The wood fiber was RAL certified. The peat substrate was produced by the company Rekyva (Lithuania) from a fraction of *Sphagnum* peat with a particle size <20 mm. The chemical composition of the wood fiber and the peat substrate was analyzed in the laboratory of the Lithuanian Agricultural Advisory Service and in the agrochemical research laboratory of the Lithuanian Research Centre for Agriculture and Forestry. The characteristics of the materials used for the cucumber-growing medium are presented in Table 3.

### 4.2. Plant Material and Growing Conditions

The cucumber selected for the study was the early parthenocarpic, hybrid cucumber (*Cucumis sativus* L.) variety “Dirigent H”, developed in the Netherlands. Cucumber seeds were sown in a 24-cut 5.5 × 6 × 6 cm plastic nursery (28 × 38 cm) on 21 May 2021. The nurseries were filled with a peat substrate for seed germination (Company Rekyva, Lithuania), consisting of a peat fraction of up to 7 mm. The substrate was enriched with 2.0 g of l^−1^ fertilizer N_14_P_16_K_18_. The pH of the germination substrate was 6. Cucumber seedlings were grown from seed in a regulated-climate chamber, RUMED 1301. The temperature of the propagation chamber was 25 °C, the humidity was 80%, and the daily light integral was 21.2 mol⋅m^−2^ d^−1^. The germinating cucumbers were watered manually as required and no additional fertilizer was applied. When the seedlings produced their second true leaf (7 June), they were transferred from the germination chamber to a regulated-climate glasshouse and planted one seedling at a time in 5 L plastic growing containers (D—23 cm (SBH)) filled with the test growing medium. The average daily temperature in the greenhouse during the whole experiment was 25 °C, the humidity was 65%, and the daily light integral was 21.8 mol⋅m^−2^ d^−1^. The cucumbers were watered every two hours. Water was supplied to each plant using a capillary irrigation system. The growing containers were protected with a polyethylene film to prevent excess water from draining into the drainage system. The plants were grown as a single stem throughout the study. Cucumbers were grown upwards using polypropylene ropes. Shoots were removed daily to maintain a single stem.

### 4.3. Plant Fertilization

The cucumbers grown in the experiment were fertilized with YaraMila^®^ COMPLEX NPK 12-11-18 mineral fertilizer with microelements. This complex fertilizer contains all the essential nutrients needed by the plant and only a small amount of chloride, and can therefore be used on chlorine-sensitive plants. The chemical composition of the fertilizer is as follows: nitrogen (N): 12%, phosphorus (P_2_O_5_/P): 11/4.8%, potassium (K_2_O/K): 18/14.9%, magnesium (MgO/Mg): 2.7/1.8%, sulfur (SO_3−_/S): 20/8%, boron (B): 0.015%, iron (Fe): 0.20%, manganese (Mn): 0.02%, and zinc (Zn): 0.02%. A total of 1.60 kg of complex fertilizer was dissolved in 1 m^3^ of water. Before flowering, the cucumbers were fertilized once a week with 500 mL of nutrient solution in the growing container. During the growing season, the cucumbers were fertilized twice a week with this fertilizer. Additional nitrogen fertilization was started 9 days after transplanting. Water-soluble calcium nitrate (Ca(NO_3_)_2_) fertilizer was used. This is a versatile nitrogen fertilizer used to fertilize calcium-demanding plants during the growing season, and is particularly suitable for more acidic soils. The chemical composition of the fertilizer is as follows: total nitrogen (N)—15.5% (nitrate nitrogen (N-NO_3_)—14.4%, ammonia nitrogen (N-NH_4_)—1.1%) and calcium oxide (CaO)—26.3% (calcium 18.8%). Calcium nitrate was applied once a week (11 times every 7 days) to the plants during the growing season to provide them with the additional nitrogen required in the experiment.

### 4.4. Experimental Design

**Figure 4:**
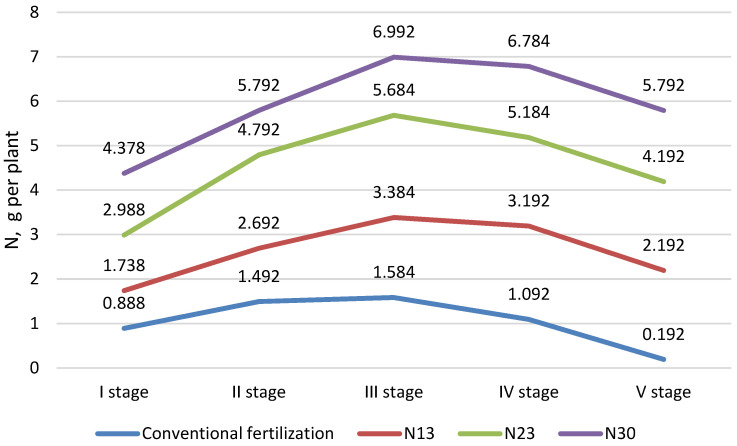
Nitrogen fertilization during the cucumber-growing season (five experimental stages), N13, N23, and N30—additional nitrogen fertilization.

The vegetative containers were arranged in rows on either side of the irrigation pipe. Each row contained 12 vegetative containers spaced 30 cm apart. The whole experiment consisted of 16 rows, i.e., 192 vegetative containers. One irrigation capillary (drip sprinkler) was introduced into each vegetative container. Four growing media (four treatments of the experiment (factor A)) were studied: (1) 100% peat (PS) (control substrate) (2 kg per container); (2) 100% wood fiber (WF) (0.8 kg per container); (3) 50/50 wood fiber/peat by volume (WF/PS 50/50) (1.7 kg per container); and (4) 25/75 wood fiber/peat by volume (WF/PS 25/75) (1.8 kg per container). Both mixtures of wood fiber and peat were prepared just before the start of the experiment (company Rekyva, Lithuania). Each treatment of the growing media (substrate) was divided into four groups of four fertilization backgrounds (factor B): (1) conventional fertilization (control); (2) N_13_ as an additional fertilization; (3) N_23_ as an additional fertilization; and (4) N_30_ as an additional fertilization. The distribution of nitrogen (N) during the cucumber-growing season is shown in Figure 4.

Each treatment consisted of 48 growing containers with one cucumber plant each. The experiment was carried out in three replications. The growing containers were arranged in a completely randomized design.

The experiment was carried out in five stages: stage I—27 days after transplanting (beginning of flowering and fruit formation), stage II—39 days after transplanting (beginning of fruiting), stage III—52 days after transplanting (peak of cucumber fruiting), stage IV—64 days after transplanting (end of cucumber fruiting), and stage V—80 days after transplanting (end of the growing season).

### 4.5. Plant Growth and Yield Measurements

The plant biometric parameters were determined at the end of stage I, at the time of flowering, and at fruit formation. The biometric and yield structure elements of each plant were determined, such as the plant height, number of leaves, and number of flowers on the plant. The chlorophyll index was determined using the OPTI-SCIENCES instrument (CCM-200 plus, measurement units: CCI—chlorophyll content index). The chlorophyll index of cucumber leaves was determined by taking five measurements on the third leaf from the top of each plant. Observations were made five times during the growing season, starting at the cucumber flowering and fruit formation stage, 21 days after transplanting.

At the beginning of cucumber fruiting, between 29 and 80 days after transplanting, cucumber productivity was determined by picking, weighing, and counting the marketable fruit from each cucumber variety (treatment) separately. Cucumber fruit was harvested twice a week. The total cucumber productivity during the growing season was determined at the end of the experiment (80 days after transplanting) by converting the results obtained into productivity in kg m^−2^.

At the end of each stage of the study, plants were cut from the two vegetative containers of each variant, and the green mass of the aboveground part was determined. The plants with all their morphological parts were weighed and crushed. The dry matter of the aboveground part of the plant was determined by drying at 105 °C in a drying oven Memmert (Germany) to a constant weight.

### 4.6. Measurement Properties of Growing Media 

The agrochemical parameters of the growing media were determined at the beginning of the experiment and five times throughout the cucumber-growing season, i.e., at the end of each stage of the experiment. Samples of the growing medium were taken from the two vegetative containers of each variant, after cutting the aboveground part of the plant, for agrochemical analyses and for the determination of dry matter content.

Organic carbon, nitrogen, phosphorus, and potassium in the growing media were determined according to standard methods. Organic carbon (C) was determined using the Tyurin spectrophotometric method [50]. The sample was heated with a solution of potassium bichromate in sulfuric acid (+160 °C). The final measurement of C at 590 nm was carried out on a Cary 60 spectrophotometer (Varian) using glucose standard solutions. The total nitrogen (N_total_) content of the soil was determined by the Kjeldahl method (the Kjeldahl apparatus Vapodest) with the spectrophotometric measurement at 655 nm. The mineral nitrogen content was determined spectrometrically using sulfosalicylic acid [51]. The total phosphorus (P) was measured at 430 nm and the total potassium (K) was measured by atomic absorption spectrometry using an AAnalyt 200 instrument after mineralization in sulfuric acid [52]. The copper (Cu) and zinc (Zn) contents were determined according to EN 13650:2003 and EN 8288:2002. The magnesium (Mg) and total iron (Fe) were determined according to EN 13650:2003, AOAC 974.27, and boron (B) was determined according to EN 13650:2003, EN ISO 11885:2009. All nutrients were determined in three replications.

The electrical conductivity (EC) of the growing media was determined with a Delta-T Devices HH2 Moisture Meter at a depth of 10 cm in each growing container six times during the growing season. The first measurement was taken on the day after the cucumbers were transplanted into the growing containers. Subsequent measurements were taken at the end of each phase of the study and at the end of the growing season.

### 4.7. Statistical Analysis

All data were statistically analyzed using the computer program package SPSS (IBM^®^ SPSS^®^ software) by the method of a two-way (growing media × fertilization) analysis of variance (ANOVA). Statistically significant differences in the data were determined by Fisher’s criterion and a least significant difference (LSD) test at the probability level of 95% (*p* ≤ 0.05). The mean error, SE, was determined by a basic statistical analysis of characteristics.

## 5. Conclusions

Wood fiber can be used as an alternative to peat substrate for growing cucumbers in greenhouses. The best results were obtained when cucumbers were grown in a wood fiber and peat mixture of WF/PS 25/75 without additional nitrogen fertilization. Increasing the amount of wood fiber in the growing media to 50% (50/50) gave better results with additional nitrogen fertilization (N_13_ and N_30_). Cucumbers grown in wood fiber media required additional nitrogen fertilization using N_30_. Wood fiber is a good substitute for peat, but it is better used in a mixture with a peat substrate. More research should be carried out on wood fiber growing media to assess the amount of nitrogen used in the microbial decomposition process and how this affects the amount and uptake of the macro- and micronutrients required by the plant, as well as the biochemical processes involved in the use of wood fiber in plant production. The response of other vegetables to the growing media of wood fiber should be determined.

## Figures and Tables

**Table 1:** The influence of four growing media: 100% peat substrate (PS), 100% wood fiber (WF), 50/50 wood fiber/peat by volume (WF/PS 50/50), and 25/75 wood fiber/peat by volume (WF/PS 25/75), and conventional fertilization (CF) or supplementary nitrogen fertilization with nitrogen N_13_, N_23_, of N_30_ on the biometric parameters of cucumber.

Growing Media	N fertilization Rate	27 Days after Transplanting					80 Days after Transplanting			
		Plant Height, cm	Number of Flowers, Units Per Plant	Number of Leaves, Units Per Plant	Above-ground Fresh Mass, g Per Plant	Dry Matter Content of the Plants, %	Number of Fruits m^−2^	Fruit Weight, kg m^−2^	Aboveground Fresh Mass, g Per Plant	Dry Matter Content of the Plants, %
PS	CF	124 a	3.0 a	13.0 a	270.5 cde	9.3 bcde	75.4 b	5.91	234.6 ab	22.56 h
	N_13_	112 abcd	2.6 a	11.0 cd*	326.9 ab*	8.7 ef*	79.4 a	6.13	215.c*	25.61 gh
	N_23_	87 e*	2.5 a	11.0 cd*	266.5 cdef	8.9 def	64.6 c	5.48	154.9 hj*	59.46 a*
	N_30_	94 def	2.6 a	12.6 ab	312.5 abc	10.2 a*	62.0 d	4.93	178.1 fgh*	24.17 gh
WF	F	66 gh*	0 e*	8.4 h*	179.6 gh*	10.6 a*	70.0 c*	6.20	171.3 gh*	36.16 d*
	N_13_	59 h*	0.2 e*	8.8 gh*	289.4 bc	9.0 cdef	76.0 b*	5.92	205.2 cd*	29.30 efg*
	N_23_	61 h*	0.4 e*	9.2 efg*	207.8 gh*	9.7 ab	76.0 b*	5.19	146.4 jk*	31.99 def*
	N_30_	67 gh*	0.4 e*	9.0 fgh*	225.4 efg	9.1 cdef	86.0 a*	5.39	165.2 h*	32.53 def*
WF/PS 50/50	F	95 adef*	1.2 bcd*	11.0 cd*	289.5 bcd	10.1 a*	78.2 b*	5.97	250.6 a*	27.40 fgh
	N_13_	93 ef*	1.0 cde*	10.6 cd*	353.8 a*	8.7 ef*	91.2 a*	6.68	185.2 efg*	31.49 def*
	N_23_	93 ef*	0.8 d*	10.4 de*	260.2 de	8.8 ef	76.4 b*	6.07	192.9 def*	34.71 de*
	N_30_	103 bcde*	1.1 cde*	11.8 abc	226.5 efg	9.4 bcd	83.6 b*	6.52	122.1 lm*	45.49 c*
WF/PS 25/75	F	90 ef*	2.4 ab	11.4 bcd*	164.2 h*	9.2 bcde	82.4 a*	6.16	195.1 de*	32.59 de*
	N_13_	92 ef*	2.1 abc	10.6 cd*	258.0 def	8.6 f*	73.4 b*	5.73	210.8 c*	26.39 fgh
	N_23_	96 cdef*	2.0 abc	10.4 cde*	158.2 h*	9.0 cdef	59.4 d*	4.86	136.4 kl*	36.52 d*
	N_30_	84 fg*	1.8 abcd	10.2 def*	219.4 fg*	9.6 bc	63.6 c*	5.18	114.3 m	51.53 b*

Note: values in the columns marked with * are significantly different from the control (peat substrate, conventional fertilization). Values marked with different letters (a, b…) in the columns are significantly different according to Fisher’s least significant difference, with *p* < 0.05. There was no interaction between factors for cucumber fruit content values, and fruit weight was not statistically evaluated.

**Table 2:** The influence of conventional fertilization (CF) and additional nitrogen fertilization with N_13_, N_23_, or N_30_ on total (N_total_%) and mineral (N_min_ mg kg^−1^) nitrogen content in four cucumber-growing media: 100% peat substrate (PS), 100% wood fiber (WF), 50/50 wood fiber/peat volume ratio (WF/PS 50/50), and 25/75 wood fiber/peat volume ratio (WF/PS 25/75). I–V—five experimental stages: 27, 39, 52, 64, and 80 days after transplanting of cucumbers into the growing media.

Growing Media	N Fertilization Rate	N_total_					N_min_	
		Experimental Stages					Stage I	Stage V
		I	II	III	IV	V		
PS	CF	1.08 a	1.17 a	1.37 a	1.48 a	1.05 a	5.1 c	113.7 b
	N_13_	1.07 a	1.16 a	1.51 a	1.39 a	1.26 a	38.8 c*	100.4 b
	N_23_	1.17 a	1.26 a	1.66 a	1.47 a	1.22 a	118.0 b*	160.2 a*
	N_30_	1.27 a	1.44 a	1.63 a	1.45 a	1.61 a	198.4 a*	171.9 a*
WF	CF	0.22 c*	0.79 a	0.53 a*	0.43 a*	0.44 b*	0.9 c	8.5 c*
	N_13_	0.59 b*	0.48 b*	0.58 a*	0.70 a*	0.89 ab	29.2 c	204.9 b*
	N_23_	0.92 a	0.87 a	0.98 a	0.70 a*	1.22 a	138.4 a*	267.6 a*
	N_30_	0.58 c*	1.12 a	0.90 a	0.65 a*	0.95 ab	145.9 a*	266.1 a*
WF/PS 50/50	CF	0.95 a	0.97 a	1.25 a	1.35 a	1.00 b	2.0 b*	19.4 c*
	N_13_	0.91 a	1.05 a	1.36 a	1.25 a	0.89 b	43.1 b*	79.7 b*
	N_23_	0.89 a	1.19 a	1.57 a	1.49 a	1.45 ab	70.2 b*	240.1 a*
	N_30_	1.08 a	1.25 a	1.39 a	1.28 a	1.6 a	151.7 a*	277.3 a*
WF/PS/25/75	CF	0.95 a	1.06 a	1.43 a	1.38 a	1.09 a	2.8 c	158.7 b*
	N_13_	1.03 a	1.13 a	1.42 a	1.61 a	1.07 a	14.2 c	236.8 a*
	N_23_	1.05 a	1.37 a	1.82 a	1.34 a	1.60 a	137.6 b*	230.2 a*
	N_30_	1.30 a	1.51 a	1.69 a	1.55 a	1.65 a*	186.8 a*	240.8 a*

Note: values marked with * in the columns are significantly different from the control (peat substrate, conventional fertilization). Values marked with different letters (a, b…) in the columns are significantly different in the media between nitrogen fertilization rates according to Fisher’s least significant difference with *p* < 0.05.

**Table 3:** Characteristics of wood fiber and *Sphagnum* peat used in the experiment as growing media.

Parameter	Wood Fiber	*Sphagnum* Peat
pH	4.6	5.3
N total% DW *	0.22	1.08
P mg kg^−1^ DW	57	181
K mg kg^−1^ DW	472	674
Mg mg kg^−1^ DW	29.3	23.2
Fe mg kg^−1^ DW	113.0	86.2
Cu mg kg^−1^ DW	2.9	3.0
Zn mg kg^−1^ DW	10.4	12.4
B mg kg^−1^ DW	15.3	14.0
Cl mg kg^−1^ DW	117.0	29.9

*—DW, the parameter has been determined in the dry weight. Wood fiber density was 0.16 g cm^−3^, air-dry moisture content was 41%, and water absorption was 2.1 g g^−1^.

## Data Availability

Not applicable.
